# Study of Optimal Conditions to Grow Thai *Ganoderma*, Fruiting Test, Proximate and Their Alpha Glucosidase Inhibitory Activity

**DOI:** 10.3390/life13091887

**Published:** 2023-09-09

**Authors:** Naruemon Wannasawang, Thatsanee Luangharn, Anan Thawthong, Rawiwan Charoensup, Wuttichai Jaidee, Wirongrong Tongdeesoontorn, Kevin D. Hyde, Naritsada Thongklang

**Affiliations:** 1 Center of Excellence in Fungal Research, Mae Fah Luang University, Chiang Rai 57100, Thailand; naruemon.wan@mfu.ac.th (N.W.); thatsanee.lua@mfu.ac.th (T.L.); anan.thawthong@outlook.com (A.T.); kdhyde3@gmail.com (K.D.H.); 2 School of Science, Mae Fah Luang University, Chiang Rai 57100, Thailand; 3 School of Integrative Medicine, Mae Fah Luang University, Chiang Rai 57100, Thailand; rawiwan.cha@mfu.ac.th; 4 Medicinal Plant Innovation Center of Mae Fah Luang University, Chiang Rai 57100, Thailand; wuttichai.jai@mfu.ac.th; 5 School of Agro-Industry, Mae Fah Luang University, Chiang Rai 57100, Thailand; wirongrong.ton@mfu.ac.th; 6 Research Group of Innovative Food Packaging and Biomaterials, Mae Fah Luang University, Chiang Rai 57100, Thailand

**Keywords:** cosmopolitan genus, optimal condition, traditional medicine, white rot

## Abstract

*Ganoderma* (*Ganodermataceae*) has a worldwide distribution and has been widely used in traditional medicines. In this study, we report wild strains of *Ganoderma* that include two *G. sichuanense* and one *G. orbiforme* from northern Thailand. Optimal conditions for mycelium growth were ensured. The most favourable medium was potato sucrose agar for *G. sichuanense* and oatmeal agar for *G. orbiforme* and at 25 °C and 30 °C and pH 4–8. All types of cereal grains can be used to promote the growth of the mycelia of *Ganoderma* species. Fruiting tests were performed. All strains of *Ganoderma* produce fruiting bodies successfully in bag culture at 28 ± 1 °C with 75–85% relative humidity. Only *G. orbiforme* produced fruiting bodies in field cultivation at the laboratory scale. In the first flush yields, the *G. sichuanense* strain MFLUCC 22-0064 gave better production (the B.E was 152.35 ± 6.98 g). This study is the first to document the bag and field cultivation of wild Thai *G. orbiforme*. *Ganoderma* species are revealed to contain high amounts of fiber (47.90–52.45% d.b.), protein (12.80–14.67% d.b.), fat (4.90–5.70% d.b.), and carbohydrates (3.16–4.02% d.b.). Additionally, *G. sichuanense* and *G. orbiforme* were preliminarily screened for biological activity for inhibition of alpha–glucosidase enzyme activity. The IC_50_ values of *G. orbiforme* (MFLUCC 22-0066) was 105.97 ± 1.36 µg/mL and *G. sichuanense* (MFLUCC 22-0064) was 126.94 ± 0.87 µg/mL. Both strains had better inhibition than acarbose (168.18 ± 0.89 µM). These results on wild strains of *Ganoderma* will be useful for further studies on the applications of *Ganoderma*. Later the species can be introduced to domestic markets for cultivation and medicinal use.

## 1. Introduction

*Ganoderma* (*Ganodermataceae*, Basidiomycota) was established by Karsten [1] with *Ganoderma lucidum* (Curtis) P.Karst. as the type species. Justo et al. [2] treated *Ganodermataceae* as a synonym of Polyporaceae, while Cui et al. [3] reported that *Ganoderma* is quite different from Polyporaceae. *Ganoderma* is distinctively characterized by laccate and nonlaccate basidiocarps, double-walled basidiospores, an apical germinal pore, thin and colourless external wall (exosporium), with a brown to dark brown internal wall (endosporium) [4,5,6]. There are 493 records of *Ganoderma* species in the Index Fungorum [7]. The morphological characteristics and molecular analysis of Thai wild *Ganoderma* have been reported [8,9,10]. Members of *Ganoderma* are cosmopolitan species [11], which are distributed worldwide in subtropical to tropical and temperate regions [12]. Some *Ganoderma* are regarded as tree pathogens [9,13,14,15] causing white rot diseases on rotting stumps, roots, and living trunks [16]. *Ganoderma* is economically important, due to the fact that members of the genus are regarded as valuable medicinal mushrooms [17,18]. Several *Ganoderma* species are known to be prolific sources of highly natural bioactive compounds such as polysaccharides, proteins, steroids, and triterpenoids [19,20,21]. These natural bioactive compounds are used to treat and remedy many pathological diseases [22,23]. In addition, *Ganoderma* products come in the form of coffee, powder, tea, dietary supplements, spore product, drinks, syrup, toothpaste, soap, lotion, and have been commercialized as effective food and drug supplements for health benefits [24,25,26].

*Ganoderma sichuanense* is recognized as a medicinal mushroom. This fungus was initially documented in the tropical region of Thailand by Thawthong et al. [10]. This delineation was described in both taxonomical classification and molecular substantiation. *Ganoderma sichuanense*, a reservoir of bioactive compounds, can address human ailments. The comprehensive exploration of this fungus, such as optimal conditions, fruiting trials, nutritional constituents, and assessing alpha-glucosidase inhibitory effects, still needs to be included in the scientific report, as does *Ganoderma orbiforme*, a member of the laccate *Ganoderma* species. Nevertheless, there is a lack of research concerning its medical utility and cultivation in Thailand.

In Thailand, there are limited studies on the cultivation of *Ganoderma* species. In an attempt to further expand our knowledge of Thai *Ganoderma* species in this study, we describe *Ganoderma sichuanense* and *Ganoderma orbiforme* using morphology and molecular data. Optimal conditions for mycelia, spawning production, proximate and alpha glucosidase inhibitory activity of these fungi are also reported. These two species are likely to be used for cultivation and medicinal purposes as a new native mushroom source in Thailand.

## 2. Materials and Methods

### 2.1. Mushroom Collections

Two *Ganoderma sichuanense* and one *G. orbiforme* were collected from Chiang Mai Province, Thailand by Anan Thawthong. The fresh basidiomata of *Ganoderma* were isolated by tissue culture technique on potato dextrose agar (PDA) medium, and incubated at 25 °C for 2 weeks for later use. The mycelium cultures were deposited at the Mae Fah Luang University Culture Collection (MFLUCC) as MFLUCC 22-0066 (*G. orbiforme*), MFLUCC 22-0064 and MFLUCC 22-0065 (*G. sichuanense*), while dried samples were deposited at Mae Fah Luang University Fungarium (MFLU) as MFLU22-0111 (*G. orbiforme*), MFLU22-0109 and MFLU22-0110 (*G. sichuanense*).

### 2.2. DNA Extraction, Polymerase Chain Reaction (PCR) and Sequencing

Dried internal tissues from the fruiting bodies were used to extract DNA using the High Pure PCR Template Preparation Kit (Roche), following the manufacturer’s instructions. Total reaction mixtures (25 μL) contained 9.5 μL ddH_2_O, 12.5 μL of PCR master mix, 1 μL of DNA template, and 1 μL of each primer (10 μM). The primers used in PCR amplification were: ITS4/ITS5 for internal transcribed spacer gene region (ITS); LROR/LR5 for partial large subunit rDNA gene region (LSU) [27,28] and bRPB2-6F/bRPB2-7.1R for partial RNA polymerase II second largest subunit gene (RPB2) [29]. PCR amplification conditions were 3 min at 94 °C, followed by 35 cycles of 95 °C for 30 s, 55 °C for 1 min, 72 °C for 1 min, followed by a final extension at 72 °C for 10 min for ITS and LSU, and 3 min at 94 °C followed by 35 cycles of 94 °C for 1 min, 59.1 °C for 2 min, and 72 °C for 1 min, followed by a final extension at 72 °C for 10 min for RPB2. The PCR products were sequenced by SolGent Co., Ltd. Daejeon, South Korea.

### 2.3. Phylogenetic Analyses

**Figure 1:**
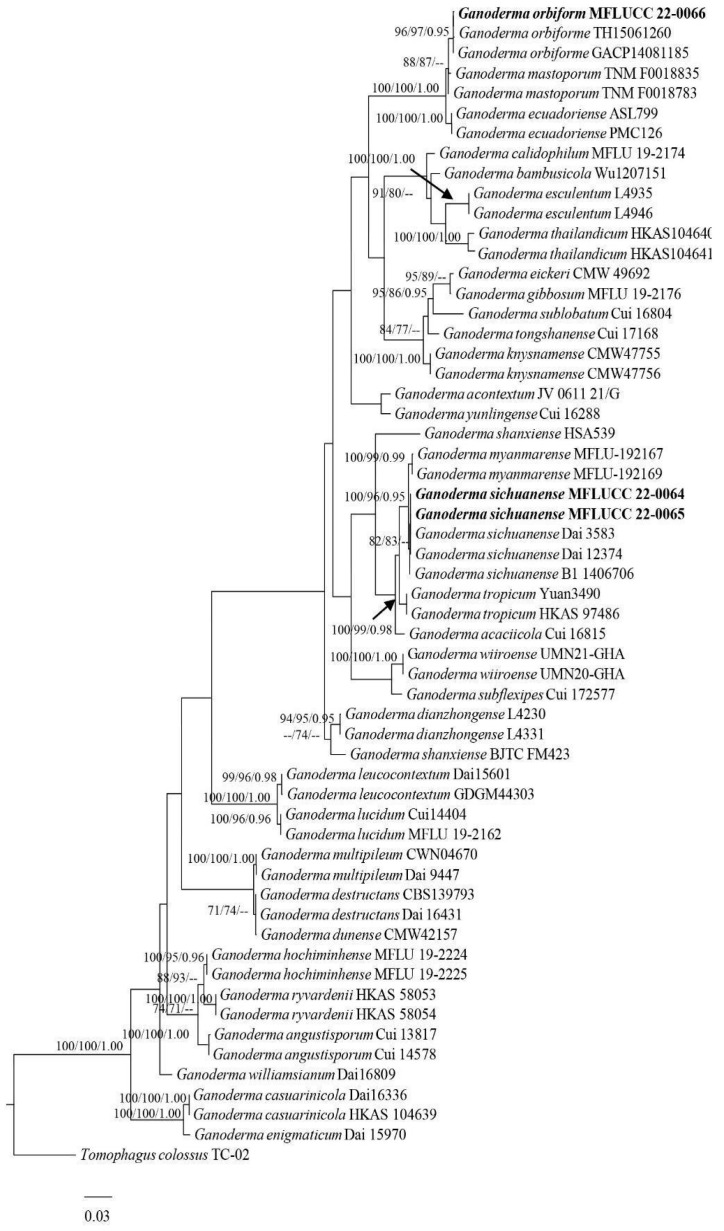
Phylogram of *Ganoderma* species obtained from maximum likelihood (RAxML) of combined ITS dataset. Bootstrap values (BS) from maximum likelihood (ML, left) and maximum parsimony (MP, middle) greater than 70% and Bayesian posterior probabilities (PP), greater than 0.95, are indicated above the nodes as MLBS/MPBS/PP. The tree is rooted with *Tomophagus colossus* TC-02. Black arrow indicates the values of MLBS/MPBS/PP of *Ganoderma tropicum*.

Sequence accession numbers in the analysis are provided in Table 1. The sequences were subjected to standard BLASTn searches in GenBank to determine the primary identity of the fungal isolates. *Tomophagus colossus* TC–02 [30] was selected as outgroup taxa. All generated sequences were aligned with the combined datasets of ITS, LSU, and RPB2 and were aligned using the MAFFT v.7.110 online program (http://mafft.cbrc.jp/alignment/server (accessed on 8 February 2023)) [31] and manually adjusted via BioEdit 7.2.3 [32]. Gaps were treated as missing data. Phylogenetic analyses were performed by using PAUP v.4.0b10 [33] for maximum parsimony (MP) and MrBayes v. 3.2.2 [34] for Bayesian analyses. Maximum likelihood analyses (ML) were estimated by using the software on the CIPRES Gateway platform [35] and performed using RAxML-HPC2 on XSEDE (v. 8.2.8) [36], then carried out using the raxmlGUI version v. 1.3.1 [37]. MrModeltest v. 2.3 was used to determine the best-fitting substitution model for each single gene partition and the concatenated dataset for Bayesian analyses [38]. Bayesian inference posterior probabilities (PP) with a GTR+I+G model was used for each partition. Phylogenetic trees were sampled every 100th generation (resulting in 10,000 total trees) in 1,000,000 generations from the running of six simultaneous Markov chains. The first 2000 trees, which contained the burn-in phase of the analysis, were discarded. The remaining 8000 trees were used to calculate the PP in the majority-rule consensus tree. ML and MP bootstrap values, equal to or greater than 70% and Bayesian Posterior Probabilities (BP) equal to or greater than 0.95 are presented above each node (Figure 1). The trees were figured in the FigTree v. 1.4.0 program [39], edited using Microsoft Office PowerPoint 2010 and exported to Adobe Illustrator CS v. 3 (Adobe Systems, San Jose, CA, USA). Sequences derived in this study are deposited in GenBank (http://www.ncbi.nlm.nih.gov, accessed on 8 February 2023).

### 2.4. Taxonomy Study

Morphological characteristics were described following the methodology described by Lodge et al. [57]. Macromorphological characteristics were examined using the Leica M125C (Leica, Wetzlar, Germany) digital microscope camera. Colors were recorded following the instructions of Kornerup and Wanscher [58]. Micromorphological characteristics were observed using a compound Nikon Eclipse Ni-E (Nikon, Tokyo, Japan) microscope. Microscopic features and measurements were made from glass slide preparations, staining tissues with 5% potassium hydroxide (KOH) and Melzer’s reagent. The features of the basidiospore, the hyphal system, the color, the sizes, shapes, and photographs were recorded and measured using the Tarosoft Image Framework programme v. 0.9.7. The size of the basidiospore was measured with and without the myxosporium using at least 50 basidiospores from each basidiomata [59]. The basidiospore quotient was followed [Q = L/W] with dimensions are given as (a—) b—c—d (—e), where Q, the quotient of basidiospore length to width (L/W) of a basidiospore inside view, and Qm, the mean of Q values ± SD, were calculated considering the mean value of the lengths and widths of basidiospores [60].

### 2.5. Optimal Agar Medium for Mycelium Growth

Five different medium types, namely potato dextrose agar (PDA), potato sucrose agar (PSA), corn meal agar (CMA), oatmeal agar (OMA), and malt extract agar (MEA) were tested for the growth of mycelium of two strains of *G. sichuanense* and one strain of *G. orbiforme*. The media plates (9 cm) were centrally inoculated with mycelium plugs of approximately 0.5 mm diam and incubated at 25 °C for 8 days in the dark. The medium was melted and washed away with hot water, leaving the fungal mycelia. The growth of the mycelium was obtained by determining the dry weight in triplicate.

### 2.6. Optimal Temperature and pH for Mycelium Growth

The best agar medium was selected to test the optimal temperature and incubated at 20, 25, 30 and 40 °C. The medium was melted, the agar was washed with hot water and the mycelium was collected. The mycelium growth was obtained by determining the dry weight on day 8 in triplicate.

The suitable pH was determined in potato dextrose broth (PDB) that was adjusted to pH 2, 4, 6, 7 and 8 with 1N HCl or 1N NaOH prior to autoclaving. Appropriate 100 mL of PDB was inoculated with the mycelium, and incubated at 25 °C on a rotary shaker at 120 rpm for 14 days. Mycelial growth was evaluated via the determination of dry weight on day 14 in triplicate.

### 2.7. Effect of Spawn Production

Five cereal grains, *Coix lacryma-jobi* (millet), *Hordeum vulgare* (barley), *Oryza sativa* (rice berry), *Triticum aestivum* (wheat) and *Zea mays* (maize), were used for the testing of *Ganoderma* species. Grains were washed and soaked overnight, then water was drained off, and grains were boiled for 15 min. Fifty-gram samples of cereal grains in test tubes (25 × 200 mm) were autoclaved at 121 °C for 15 min and left at room temperature to cool. The three mycelial plugs (approximately 0.5 cm diam) were inoculated into test tubes and incubated at 25 °C. Linear mycelium length was measured for 18 days. The experiment was determined in five replicates.

### 2.8. Fruiting Test of Thai Wild Ganoderma in Bags and Field Cultivation

For bag cultivation, rubber sawdust was used as the main substrate and mixed with supplements following the instructions of Thongklang et al. [61]. Fifty grams of spawn was inoculated in bags and incubated at 25 ± 1 °C in the dark for 110 days. The same temperature and 75–85% relative humidity were used for the fruiting stage. The experiment was determined in 15 replicates. Field cultivation was conducted on the farmland of Mae Fah Luang University. The plot size was 3 m × 1 m for five replicates for each strain. The inoculated sawdust bags were inoculated and covered by soil casing (3 cm thick). During the experiment, relative humidity was maintained at 70% by watering 2 times per day for 170 days.

### 2.9. Statistical Analysis

Mycelial growth rate in media, temperature, pH, and spawning production of mushroom strains was determined and data were statistically analyzed in terms of variance of means using Duncan’s test with significance for *p* < 0.05.

The fruiting bodies of wild Thai *Ganoderma* from both bag and field cultivations were manually harvested, counted, and weighed daily. The total weight of fresh mushrooms per spent substrate was used to calculate yield data [62,63]. In addition, biological efficiency (B.E.) was conducted using the formula: weight of harvest/weight of dry substrate) × 100% [61,64,65].

### 2.10. Proximate Analysis

Total protein, fat, fiber, and carbohydrates were calculated from the oven-dried powder using standard protocols. The total protein was calculated using the Kjeldahl method. Copper sulfate: potassium sulfate (1:10) was used for the Kjeldahl digestion catalyst. Mushroom samples (0.2–0.5 g) were placed in digestion tubes. Five grams of catalyst was added to the appropriate volume (12–15 mL) of sulfuric acid to each tube with the sample. Then, the sample was digested using VELP Scientifica S30100210 Model DKL 20 Automatic Kjeldahl Digestion (VELP Scientifica, Usmate Velate, Italy) at 420 °C for 45 min. Distillation and titration via the fully automatic Kjeldahl Analyzer–distillation unit with integrated colorimetric titrator were executed and then the percentages of N and protein were calculated automatically. Determination of the fat content was evaluated via the FOSS–Fat Analyzer–Soxtec™ 8000 (FOSS, Hillerød, Denmark). The samples (2–3 g) were put in the thimble and then placed in a Soxhlet extractor. Then, approximately 70 mL of petroleum ether was added using a dispenser. After the program was finished, the cup was heated in the oven at 105 ± 2 °C for 2 h, cooled in the desiccators, and weighed. The percentage of fat was calculated using the following equation:

% Fat = [(wt of cup + fat) − wt of cup]/wt of wet sample × 100

Determination of crude fiber was carried out using Fibertherm (Gerhardt GmbH & Co. KG, Königswinter, Germany). The fiber bag was dried at 105 ± 2 °C for 1 h and then cooled to room temperature in desiccators and checked weight (M1). Then, one gram of sample was weighed on fiber bags (M2). Fiber bags with glass spacers were put into the carousel (M1 = fiber bag without sample). Samples were defatted at a fat content of >10% with acetone or petroleum ether. Then, the fibertherm process was initiated. Subsequently, dry fiber bags were placed in the crucible at 105 ± 2 °C until the mass was constant (at least 4 h) and were cooled in desiccators and weighed (M3). The amount of ash from the sample was determined by drying in the crucible at 525 ± 25 °C for 4 h and cooled down in the desiccator for 30 more minutes. The crude fiber content (%) was calculated from the weight of the ash residue (M4), weight of the sample, and fiberbag via the following equation:

% Crude fiber = (M3 − M1 − M4)/M2 × 100

where

M1 = Weight of fiber bags without sample

M2 = Weight of dry sample

M3 = Weight of crucible and fiber bags after drying at 105 ± 2 °C for at least 4 h and cooling in desiccators

M4 = Weight of crucible and fiber bags after ashed at 525 ± 25 °C for 4 h and cool off in desiccators

Furthermore, the carbohydrate content was estimated from the formula: 100 − (ash + crude fat + crude fiber + protein) The experiment was determined in three replicates.

### 2.11. The α-Glucosidase Inhibitory Assay

The α-glucosidase inhibitory assay procedure followed a previously described method with modifications. Sample solutions at 200 μg/mL were dissolved with 10% dimethyl sulfoxide (DMSO) in phosphate buffer (pH 6.8), and then 50 μL of each sample was pipetted and mixed with 100 μL α-glucosidase enzyme (0.35 U/mL) in an Eppendorf tube. After preincubation at 37 °C for 10 min, 100 μL of 1.5 mM p-NPG was added, and the samples were further incubated at 37 °C for 20 min. Next, 1000 μL of Na_2_CO_3_ (1 M) was added to terminate the reaction. Acarbose was used as a positive control. The absorbance was measured at 405 nm with a microplate reader (PerkinElmer, Inc., Waltham, MA, USA).

## 3. Results

### 3.1. Phylogenetic Analyses

Phylogenetic analyzes included 58 taxa, and the tree was inferred from the combination of ITS, LSU, and RPB2 sequences, which comprises 2553 characters with gaps. The maximum parsimonious dataset consisted of 2103 constant, 313 parsimony-informative, and 137 parsimony-uninformative characters. Tree topologies of the ML and MP were similar to the Bayesian analysis. The final ML optimization likelihood value of −14,158.684850. Estimated base frequencies were as follows: A = 0.225017, C = 0.242278, G = 0.264931, T = 0.267773; substitution rates AC = 1.209773, AG = 3.527855, AT = 1.594973, CG = 1.560722, CT = 5.400917, GT = 1.000000.

### 3.2. Taxonomy

**Figure 2:**
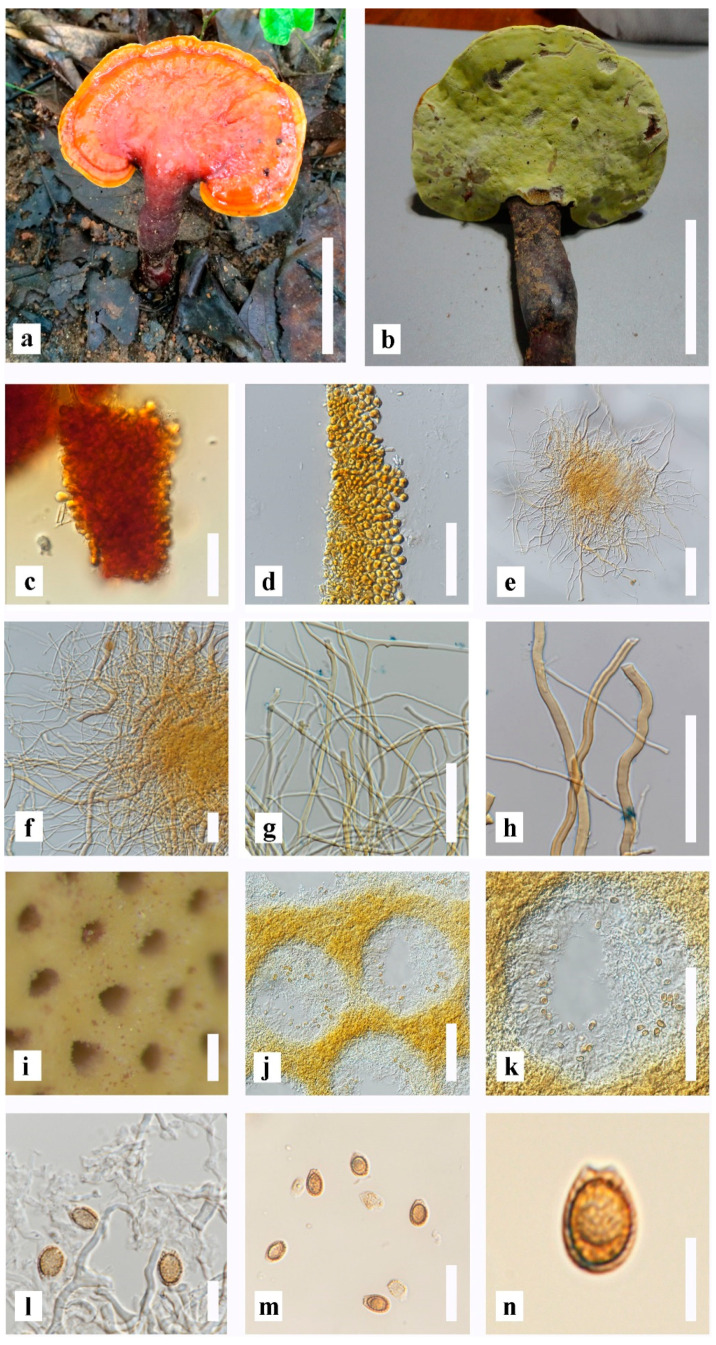
Morphological characteristics of *Ganoderma sichuanense* (MFLUCC 22-0064) from the artificially cultivated: (**a**) mature fruiting body with upper surface; (**b**) mature fruiting body with lower surface; (**c**) upper surface under Pileipellis; (**d**) Pileipellis; (**e**–**h**) context hyphae; (**i**–**k**) pores; (**l**) basidiospores with pore hyphal system; (**m**,**n**) basidiospores. Scale bars: (**a**,**b**) = 3 cm, (**c**,**d**,**f**,**h**) = 50 μm, (**e**,**j**,**k**) = 100 μm, (**g**,**l**,**m**) = 30 μm, (**i**) = 200 μm, (**n**) = 10 μm.

*Ganoderma sichuanense* J.D. Zhao and X.Q. Zhang [66] (Figure 2).

Basidiome annual, stipitate to short stipitate, laccate to strongly laccate, and woody. Pileus 5–7 × 3–10 cm, up to 1.8 cm thick at the base, suborbicular to orbicular, convex, and flabelliform shape. The surface of the pileus is reddish orange (7B8) when fresh, reddish brown (9E7) when dried, and an irregularly ruptured crust overlying the context. Context up to 1.4 cm thick, dried, brown (6E4), reddish-brown (9E7), and dark brown (7F8) at upper layer, corky when dried. Margin soft when fresh, wavy, white when become, orange (6B8) to reddish-yellow (4B7) to concolous with the pileus. Hymenophore up to 18 mm long, indistinctly stratose. Pores 4–6 per mm, round, angular, up to 119–169 × 123–191 µm ($\bar{x}$ = 146 × 158 μm, *n* = 50). Pore surface yellow when fresh, turning yellowish-white (3A2) to yellowish–grey (3B2), dull-yellow (3B3-4) when dried, and greyish-brown (7D3) when touched. Stipe 1–3 cm, lateral, some sub-cylindrical to cylindrical, thick, short, laccate, reddish-brown (9E7), concolorous with the pileus. Tubes up to 0.3–5.9 mm in length, brown (7E8). Basidiospores ellipsoid to broadly ellipsoid, some globose and ovoid, truncate at the apex with double wall (ganodermoid), brown (6E5), with a dark brown (7F8) eusporium bearing thick echinulae, (7.9–) 9.0–10.4 (–11.4) × (5.6–) 6.1–7.0 (–7.5) μm ($\bar{x}$ = 9.7 × 6.5 μm, *n* = 50) with Q = 1.2–1.6 μm, L = 9.7, W = 6.5 (including myxosporium), (5.0–) 5.8–7.7 (–8.6) × (3.2–) 4.0–5.1 (–5.6) μm ($\bar{x}$ = 6.7 × 4.5 μm, *n* = 50) with Q = 1.1–1.7 μm, L = 6.7, W = 4.5 (excluding outer myxosporium). Pileipellis a hymeniderm, brownish orange (6C8), clavate-like cells, with dextrinoid. Hyphal system trimitic; generative hyphae 1.9–2.7–3.5 μm, *n* = 20 in width, thin-walled and hyaline; skeletal hyphae 2.3–3.5–4.9 μm, *n* = 20 in width, thick–walled, branched, brownish orange (6C8); binding hyphae 1.7–3.7–5.6 μm, *n* = 20 width, thick-walled, branched, light orange (5A5) to light brown (5D5) in Melzer’s reagent.

Specimen examined: THAILAND, Chiang Mai Province, 13 July 2017, MFLUCC 22-0064, MFLU22-0109, original K17-55 and MFLUCC 22-0065, MFLU 22-0110, original K17-69.

**Figure 3:**
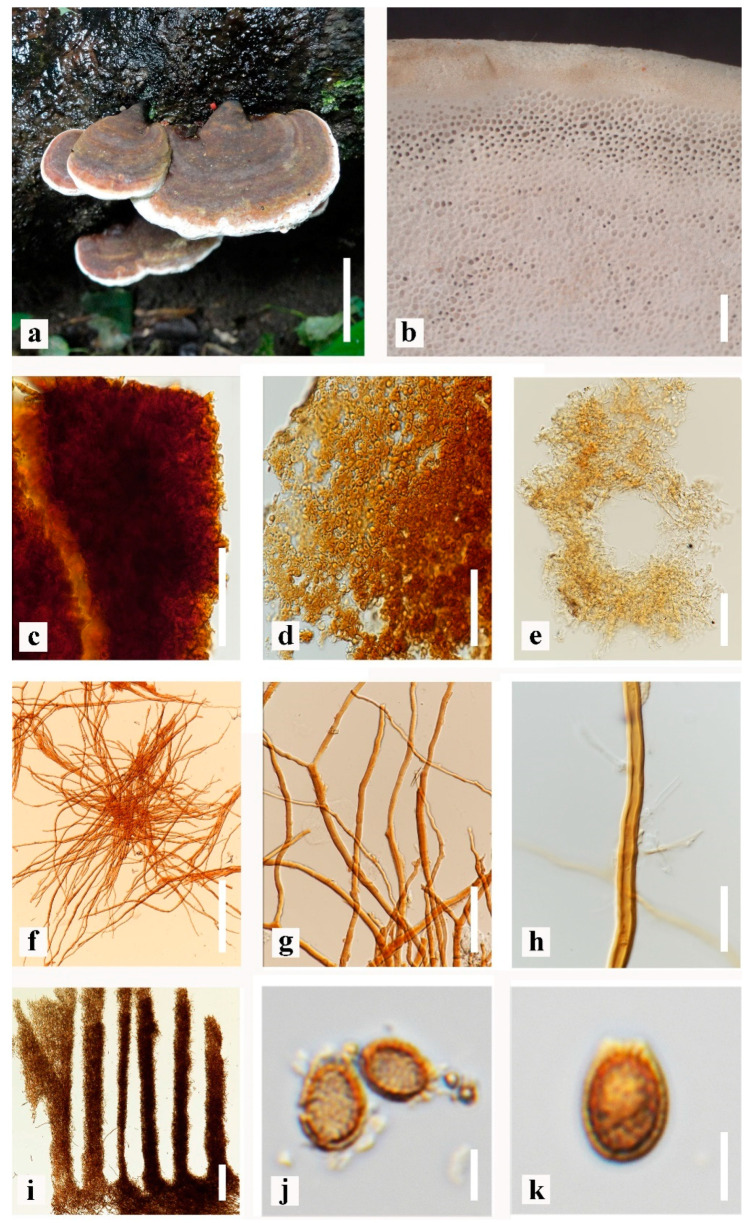
Morphological characteristics of *Ganoderma orbiforme* (MFLUCC 22-0066) from the artificially cultivated. (**a**) Mature fruiting body with upper surface, (**b**) pore characteristics, (**c**) upper surface under Pileipellis, (**d**) Pileipellis, (**e**) sections of pores, (**f**–**h**) context hyphae, (**i**) sections of tubes layers, (**j**,**k**) basidiospores. Scale bars: (**a**) = 3 cm, (**b**) = 1000 μm, (**c**) = 500 μm, (**d**,**g**) = 50 μm, (**e**) = 100 μm, (**f**) = 200 μm, (**h**) = 30 μm, (**i**) = 300 μm, (**j**,**k**) = 5 μm.

*Ganoderma orbiforme* (Fr.) Ryvarden [5] (Figure 3).

Basidiome annual to perennial, distinctly contracted base, weakly laccate, sessile, and woody when dried. Pileus 4–6 × 5–9 cm, up to 2.6 cm thick at the base, applanate to plano-convex shape with several thick layers. Pileus surface weakly laccate, smooth when young, reddish-brown (9F6-7) at concentrically zones, reddish brown (8F6) to dark brown (8F5) when dried, crust overlies the pileus, brown (7E7-8) when fresh, reddish brown (8E7–8) when dried, concentrically sulcate zones with turberculate bumps and rivulose depressions, differentiated zone at the point of attachment. Margin 1.5–3 mm, undulate and irregularities, reddish brown (8E7), soft, round, white (8A1) to orange-white (5A2) when young, brown (7D8) when become mature, and reddish brown (8E8) when dried. Hymenophore up to 2.8 mm in length with orange-grey (6B2). Pores 4–6 per mm, up to 102.6–132.8 × 79.4–131.7 µm ($\bar{x}$ = 116 × 102 μm, *n* = 50), with subcircular to circular. Pore surface white (8A1) when fresh, light brown (6D5) when touched, and turning orange-grey (5B2) when dried. Context up to 2 cm thick, soft, brown (6E8) to dark brown (6F8) at the lower layer near the tube layers, and composed of coarse loose fibrils. Tubes up to 0.1–5.6 mm in length with brown (7E5) to reddish brown (8E4) when mature. Basidiospores ellipsoid to oblong ellipsoid, some elongate with double wall (ganodermoid) at maturity, almost colorless, yellowish-brown (5D8), (8.4–) 9.8–10.9 (–11.3) × (4.4–) 5.4–6.3 (–6.6) μm ($\bar{x}$ = 10.3 × 5.8 μm, *n* = 50), with Q =1.4–2.3 μm, L = 10.3, W = 5.8 (including myxosporium), (6.1–) 7.2–8.5 (–9.2) × (3.3–) 3.7–4.6 (–5.1) μm ($\bar{x}$ = 7.9 × 4.1 μm, *n* = 50) with Q = 1.4–2.4 μm, L = 7.9, W = 4.1 (excluding outer myxosporium), yellowish brown (5D8), overlaid by a hyaline myxosporium. Pileipellis a hymeniderm, composed of apically acanthus like branched cells with some dextrinoid, with brown (6E8) to dark brown (7F8). Hyphal system trimitic; generative hyphae 2.0–3.3–4.5 μm, *n* = 20 in width, thin–walled or occasionally, hyaline, occasionally with irregular cuticle cells; skeletal hyphae 3.2–5.4–6.8 μm, *n* = 20 in width, light brown (5D8, 6D5), thick–walled; binding hyphae 3.5–4.8–6.9 μm, *n* = 20 in width, light brown (5D8), some brown (6D8), thick–walled, branched, some intertwined skeletal hyphae.

Specimen examined: THAILAND, Chiang Mai Province, 14 July 2017, MFLUCC 22-0066, MFLU22-0111, original K17-76.

### 3.3. Optimal Conditions for The Growth of The Mycelium of Wild Ganoderma from Thailand

*Ganoderma* mycelium, *G. sichuanense* (MFLUCC 22-0064 and MFLUCC 22-0065) and *G. orbiforme* (MFLUCC 22-0066) grow well in all mediums, however, the most favorable medium was potato sucrose agar (PSA) for *G. sichuanense* (both strains). The average dried weight of mushroom strains MFLUCC 22-0064 and MFLUCC 22-0065 were 0.1241 ± 0.0029 g and 0.1414 ± 0.0217 g, respectively, while oatmeal agar (OMA) was suitable for growing *G. orbiforme* (MFLUCC 22-0066). The average dried weight was 0.1496 ± 0.0118 g on day 8 after inoculation.

The optimal temperatures of these three *Ganoderma* strains are between 25 and 30 °C. *G. sichuanense* (MFLUCC 22-0064) grew well at 30 °C and followed by 25 °C, and the average dry weight was 0.1295 ± 0.0611 g and 0.0861 ± 0.0192 g, respectively. *G. sichuanense* (MFLUCC 22-0065) grew well at 30 °C, and the average dry weight was 0.1721 ± 0.0235 g. In addition, *G. orbiforme* (MFLUCC 22-0066) also prefers both temperatures (25 and 30 °C), and the average dry weight was 0.0707 ± 0.0246 g at 25 °C and 0.0512 ± 0.0006 g at 30 °C, respectively.

All pH values (4–8) were suitable for promoting mycelium growth of *G. sichuanense* (MFLUCC 22-0064 and MFLUCC 22-0065), while pH levels 4–6 were suitable for growing *G. orbiforme* (MFLUCC 22-0066) Table 2.

### 3.4. Effect of Spawn Production

The data for mycelium growth on different spawn media were investigated and is shown in Table 2. We found that all cereal grain types can be used to promote the mycelial growth of the *Ganoderma* species studied here. All types of grains can be used to grow *G. sichuanense* MFLUCC 22-0065, while *G. sichuanense* (MFLUCC 22-0064) and *G. orbiforme* (MFLUCC 22-0066) can grow in *C. lacryma-jobi*, *H. vulgare*, *O. sativa*, and *T. aestivum*. However, the mushroom grew very slowly in the cereal grain of *Z. mays*.

### 3.5. Fruiting Test of Thai Wild Ganoderma in Bags and Field Cultivation

**Figure 4:**
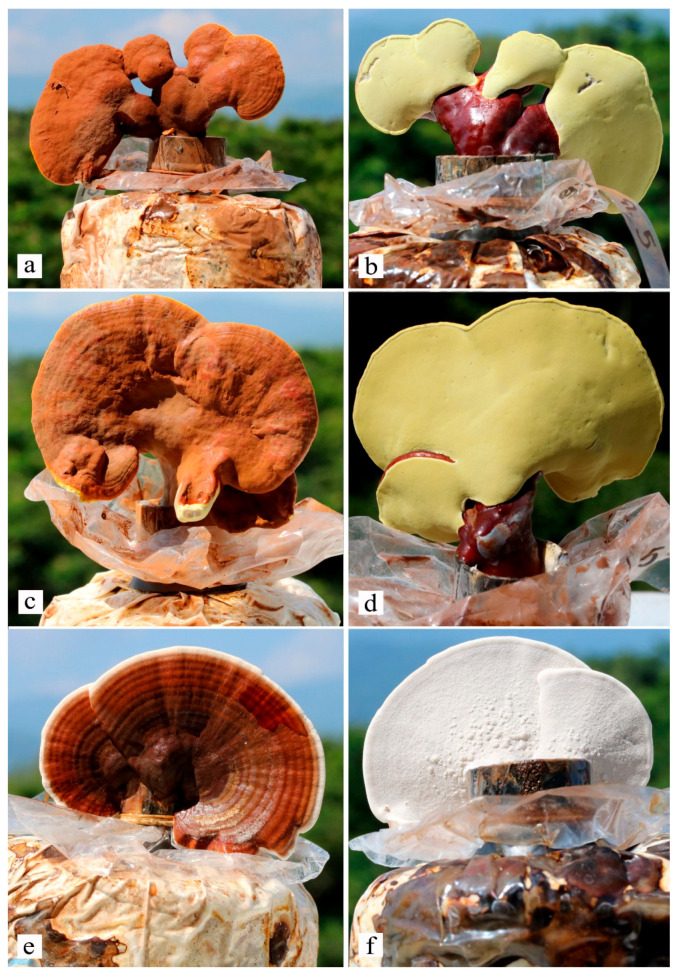
First cultivation of *Ganoderma* species from Thailand at 28 ± 1 °C in 75–85% humidity. *Ganoderma sichuanense* MFLUCC 22-0064 (**a**,**b**), *G. sichuanense* MFLUCC 22-0065 (**c**,**d**) and *Ganoderma orbiforme* MFLUCC 22-0066 (**e**,**f**).

The fruiting bodies of *G. sichuanense* (MFLUCC 22-0064 and MFLUCC 22-0065) and *G. orbiforme* (MFLUCC 22-0066) were manually harvested and measured daily. The mushrooms were produced at 28 ± 1 °C in 75–85% humidity. The cultivation of a wild strain of *G. sichuanense* (MFLUCC 22-0064 and MFLUCC 22-0065) was carried out with fifteen replicates. We found that three strains of *Ganoderma* can produce fruiting bodies on a laboratory scale (Figure 4). The mycelium of *G. sichuanense* (MFLUCC 22-0064) fully covered the rubber sawdust bags on day 31. The first primordia appeared on 44–61 days. In *G. sichuanense* (MFLUCC 22-0065) it took 22 days to fully cover the rubber sawdust bag, and the first primordia appeared on day 35–62 days. The average yield of MFLUCC 22-0064 and MFLUCC 22-0065 were 29.43 ± 4.72 g and 33.92 ± 6.98 g in the first flush production, respectively. A strain, *G. orbiforme* (MFLUCC 22-0066) was successfully cultivated in rubber sawdust bags. The mycelium was fully colonized with the bags on day 22. The first primordia appeared on day 41–58 days. The average yield was 16.21 ± 3.51 g. The yield data and the biological efficiency of three *Ganoderma* strains are given in Table 3.

**Figure 5:**
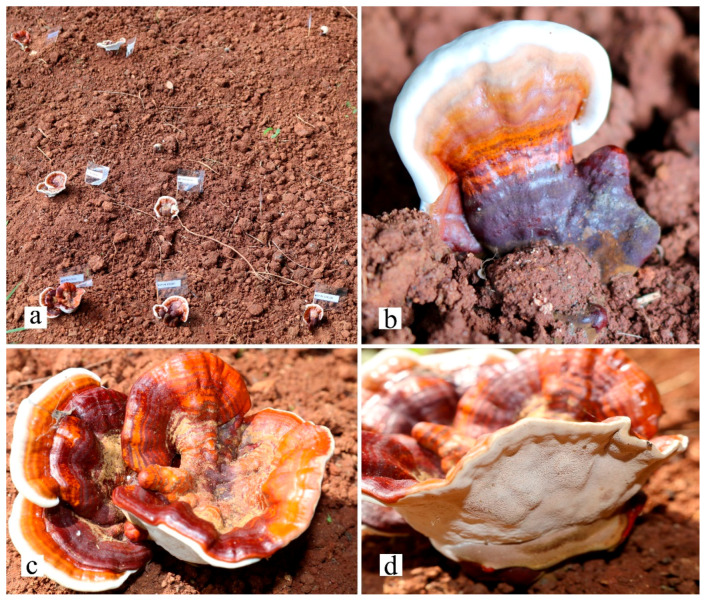
First field cultivation of wild Thai *Ganoderma orbiforme* (MFLUCC 22-0066) (**a**–**d**).

Meanwhile, the mycelium of these three strains was inoculated into a sawdust-based medium. After that, the media were fully covered by mycelia, and the bags were placed into the field and covered by soil. After 57–112 days, the first primordia of *G. orbiforme* (MFLUCC 22-0066) occurred (Figure 5). However, yield production of the strain was low. Unfortunately, *G. sichuanense* (MFLUCC 22-0064 and MFLUCC 22-0065) fruiting bodies were not produced in field cultivation.

### 3.6. Nutrition Values of Thai Wild Ganoderma

In this study, the total protein content of *Ganoderma* species varies between 12 and 15 g/100 g of sample. The highest protein content for *G. orbiforme* (MFLUCC 22-0066) was 14.67 ± 0.25% of the sample and the lowest was *G. sichuanense* (MFLUCC 22-0064) 12.80 ± 0.15%. We found that the amount of carbohydrates contained within *Ganoderma* species is 3–5% of. The highest composition of these three *Ganoderma* species is fiber. The crude fiber was found to be between 47 and 53% of the fruiting bodies. The heightened fiber was from *G. orbiforme* (MFLUCC 22-0066) (52.45 ± 0.18%), while two strains of *G. sichuanense* (MFLUCC 22-0064 and MFLUCC 22-0065) were 48.61 ± 0.67% and 47.90 ± 0.39%, respectively. In addition, the amount of fat was between 4 and 6% in the fruiting bodies of *Ganoderma* species. The fat content in *G. sichuanense* (MFLUCC 22-0064 and MFLUCC 22-0065) was 5.70 ± 0.56% and 5.11 ± 0.11%, respectively, while in *G. orbiforme* (MFLUCC 22-0066) it was 4.90 ± 0.56% (Table 4).

### 3.7. The α-Glucosidase Inhibitory Activity

The mixed fresh mushroom *Ganoderma sichuanense* (MFLUCC 22-0064 and MFLUCC 22-0065) and *G. orbiforme* (MFLUCC 22-0066) were extracted with ethanol and water under sonication for 1 h at 60 °C. The ethanol extracts were obtained by evaporation under reduced pressure, producing a yield of 2.29, 1.86, and 1.61%, respectively; water extracts of 0.55, 2.70, and 0.31% were obtained by freeze-drying. The mycelium mushrooms MFLUCC 22-0064, MFLUCC 22-0065, and MFLUCC 22-0066 were extracted with acetone and water under maceration for 24 h. Acetone extracts were obtained by evaporation under reduced pressure, to produce a yield of 2.15, 0.15, and 1.25%, respectively. The water extracts were then freeze-dried into powder. The IC_50_ value of Thai *Ganoderma* extract α-glucosidase inhibitory activity is shown in Table 5. All ethanolic extracts from fruiting bodies of three *Ganoderma* strains demonstrated that the IC_50_ ranged from 105.97 ± 1.36 to 171.68 ± 2.78. In addition, only the water extract of *G. orbiforme* (MFLUCC 22-0066) showed an inhibitory effect, and the IC_50_ value was 124.40 ± 3.18.

## 4. Discussion

*Ganoderma* has a long history of use as a traditional medicine in Asian countries. However, *Ganoderma* has been considered a very difficult genus to classify. *Ganoderma* is currently in a state of taxonomic chaos, since they were initially classified on the basis of their morphological characteristics. Based on this taxonomy and phylogenetic analysis of three *Ganoderma* strains in this study, two strains are considered to be *G. sichuanense* and another one is *G. orbiforme*. Two strains of *G. sichuanense* are clustered together with other *G. sichuanense* collections, while *G. orbiforme* is closer to the collections of *G. mastoporum* Lloyd [67], recognized *G. mastoporum* (=*G. orbiforme*) as a distinct species with lateral or dorsally lateral stipes, which stipe development varies with different growing environments [68]. Wang et al. [50] concluded that the morphological and molecular data of *G. mastoporum* are conspecific with *G. orbiforme*, and the latter is the earliest valid name for use. *Ganoderma cupreum*, *G. fornicatum*, *G. mastoporum*, *G. orbiforme*, *G. subtornatum*, and the Chinese species described; *G. densizonatum* and *G. limushanense* are morphologically conspecific with *G. orbiforme*. In this study, we report on Thai *G. orbiforme* with its molecular and morphological evidence for the first time. Morphological characteristics of *G. orbiforme* are similar to the collections that described by Ryvarden [6] and Hapuarachchi et al. [51].

It is estimated that more than 650 mushroom species are edible; however, only nine species of *Ganoderma* species are cultivated and include *G. applanatum*, *G. australe*, *G. curtisii*, *G. lucidum*, *G. oregonense*, *G. resinaceum*, *G. sinense*, *G. tenus*, *G. tropicum*, *G. tsugae* [9,69]. As *G. lucidum* is not common in nature, the number of wild mushrooms is not sufficient for commercial exploitation. Its cultivation on solid substrates, stationary liquid medium or submerged cultivation has become essential to meet the increasing demands for this mushroom. The annual sale of products derived from *G. lucidum* is estimated to be more than USD 2.5 billion in Asian countries, including China, Japan, and South Korea. Mushroom growing is also becoming increasingly popular in Thailand. *Ganoderma lucidum* has a market price of USD 33.0–50.0 per 1 kg as a commercial mushroom in Thailand. In the cultivation trials of this study, the optimal medium for *G. sichuanense* (MFLUCC 22-0064 and MFLUCC 22-0065) was PSA. Oatmeal agar was suitable to grow *G. orbiforme* (MFLUCC 22-0066). Luangharn et al. [8] reported that *Ganoderma australe* was found in Thailand. The optimal conditions of mycelia growth were found in the PDA medium. Furthermore, ref. [9] also reported that PDA, MEA, and YPD are suitable for growing *G. tropicum*. Thus, we can consume *Ganoderma* mushrooms, which can be cultivated on several substrates, depending on the species. Various cultivated mushrooms have different optimal temperatures; for example, *Volvariella volvacea* at 35 °C, *Pleurotus eryngii* at 25 °C *Phlebopus portentosus* at 30 °C [70,71] while the temperature for optimal growth of *Ganoderma* species is between 25 and 30 °C. The three strains *G. sichuanense* (MFLUCC 22-0064 and MFLUCC 22-0065) and *G. orbiforme* (MFLUCC 22-0066) grow well between 25 and 30 °C, the same as *G. australe* [8], *G.lucidum* [72], and *G. tropicum* at 25–28 °C [9].

Mushroom spawn substrates can be cereal grain, sawdust, wood chips, or rope [73,74]. The most common materials are cereal grains. Sorghum was used as the medium for all types of mushrooms [69,75]. Rye was used to grow *Agaricus* sp. [63,69]. In the case of *Ganoderma* mushrooms, durum wheat, sorghum, and wheat grain could be used as an alternative for spawn production [8,76,77,78]. In this study, we found that all types of cereal grains (barley, maize, millet, rice berry, wheat) can be used as spawn production. Similarly, Luangharn et al. [8] reported that barley, corn cobs, pearl mille, and sorghum could be used to promote the mycelium growth in spawn production. In the current trend, agricultural wastes were alternative ways to used. It can be utilized as substrates for spawning to decrease the price of mushrooms. Rashad et al. [79] grew the mycelia of *Ganoderma* in different types of agriculture waste, e.g., broad bean stalks, cotton stalk, maize straw, rice straw, sugarcane bagasse, and wheat straw mixed with wheat bran or corn gluten, and found that cotton stalk sugarcane bagasse rice straw and wheat straw showed the best supplementation for spawn production.

In cultivation trials, three strains of wild Thai *Ganoderma*, *G. sichuanense* (MFLUCC 22-0064 and MFLUCC 22-0065), and *G. orbiforme* (MFLUCC 22-0066) can be grown in rubber sawdust bags and artificially produce the fruiting bodies. Our findings are similar to those of Luangharn et al. [8], who reported that wild Thai *Ganoderma australe* produced fruiting bodies in rubber sawdust bags at 30 °C, with 60–75% humidity. However, our strains have production at 75–85% humidity. Rubber sawdust is commonly used and is the preferred medium on the commercial scale in Thailand [69]. However, the availability of raw materials of any region is a key factor when choosing agricultural waste for growing the mushrooms. Luangharn et al. [77] noted that Alnus cremastogyne sawdust that is planted in China can be used to grow three species of *Ganoderma*: *Ganoderma leucocontextum*, *G. resinaceum*, and *G. gibbosum*. Ozcariz-Fermoselle et al. [78] reported that pecan nut waste (*Carya illinoinensis*) can be used for *G. lucidum* cultivation in Spain. An important piece of further study is to find local agricultural waste substrates to grow mushrooms that can produce a better yield than sawdust alone in Thailand. As the use of local agricultural waste substrates will be shown as an environmentally friendly alternative, it might increase the benefits of local agricultural waste recycling.

Mushrooms have been used by mankind as a nutritional food. To date, in terms of nutrition, studies from mushrooms are increasing in interest. The demand for functional foods with high nutritional and medicinal value is a hot issue. Mushrooms are a good source of nutritional value. They contain high levels of protein, high fiber, carbohydrates, minerals, vitamins, and have a low fat content [50,80]. *Ganoderma* is treasured as a functional food. They are found to be a good source of nutrition component. In this study, nutrition of content of wild Thai *Ganoderma*, *G. sichuanense* and *G. orbiforme* were studied. These mushrooms revealed high amounts of fiber (47.90–52.45%), followed by the total protein (12.80–14.67%), fat (4.90–5.70%) and carbohydrates (3.16–4.02%). While Singh et al. [81] report a proximate analysis of four species of *Ganoderma* (*G. applanatum*, *G. brownie*, *G. lucidum*, and *G. philippii*) and found that those strains contained the highest carbohydrates (75.5–81.4%), proteins (9.29–13.3%), fiber (4.92–8.07%), and a low fat content (1.62–2.87%).

The α-glucosidase inhibitor has been used as a treatment for type 2 diabetes (T2D) [82]. *Ganoderma* species have an important economic value due to their medicinal properties and pathogenicity [17,83]. The genus has been used as a medicinal mushroom for a very long time. There are many reports showing α-glucosidase inhibitory activities from *Ganoderma* species such as *Ganoderma hainanense*, *G. leucocontextum*, *G. lucidum*, and *G. resinaceum* [82,84,85,86,87]. In this study, the detection of α-glucosidase inhibitory activity in Thai *Ganoderma* was found. The fruiting bodies of *Ganoderma* sp. that were extracted by EtOH showed α-glucosidase inhibitory activity. *Ganoderma orbiforme* (MFLUCC 22-0066) and *G. sichuanense* (MFLUCC 22-0064) had stronger inhibition than the positive control (acarbose). The IC_50_ values were 105.97 ± 1.36, and 126.94 ± 0.87 µg/mL, respectively. In addition, the extraction of *Ganoderma orbiforme* (MFLUCC 22-0066) by water was also successful. The IC_50_ values were 124.40 ± 3.18 µg/mL, while the acarbose was 168.18 ± 0.89 µM. These preliminary results suggest that the strains of Thai *Ganoderma* sp. in this study could be further researched and developed. 

## 5. Conclusions

In this study, the most favorable medium was potato sucrose agar (PSA) for *G. sichuanense* and oatmeal agar (OMA) for *G. orbiforme*. The suitable temperature and pH of the three strains were 25 °C and 30 °C and pH was 4–8. The most favorable spawn were rice berry, wheat, and barley, respectively. The nutritional value of three strains of wild Thai *Ganoderma* revealed high amounts of fiber and was followed by total protein. Furthermore, *G. orbiforme* (MFLUCC 22-0066) and *G. sichuanense* (MFLUCC 22-0064) showed better inhibition than acarbose. The result of this study is useful as basic information for preventing and treating diabetes. Therefore, these local *Ganoderma* strains could be industrially cultivated and introduced to the Thai market. However, more research is needed to find local agricultural waste to increase production yields and more research is needed to study glucose uptake in cell lines and clinical trials.

## Figures and Tables

**Table 1:** Taxa used in this study and their GenBank accession numbers for ITS DNA sequence data.

Fungal Species	Voucher	GenBank Accession No.			References
		ITS	LSU	RPB2	
*Ganoderma acaciicola*	Cui 16815	MZ354895	MZ355005	MZ245384	[40]
*G. acontextum*	JV 0611 21G	KF605667	-	-	[41]
*G. angustisporum*	Cui 13817	MG279170	-	-	[41]
*G. angustisporum*	Cui 14578	MG279171	-	-	[41]
*G. bambusicola*	Wu1207-151	MN957781	-	LC517944	[42]
*G. calidophilum*	MFLU 19-2174	MN398337	-	-	[43]
*G. casuarinicola*	Dai 16336	MG279173	-	-	[41]
*G. casuarinicola*	HKAS 104639	MK817650	MK817654	MK840868	[9]
*G. destructans*	CBS 139793	NR 132919	-	-	[44]
*G. destructans*	Dai 16431	MG279177	-	MG367512	[41]
*G. dianzhongense*	L4230	MW750236	-	-	[45]
*G. dianzhongense*	L4231	MW750237	-	MZ467043	[45]
*G. dunense*	CMW42157	MG020255	-	-	[46]
*G. ecuadoriense*	ASL799	KU128524	KX228350	-	[47]
*G. ecuadoriense*	PMC126	KU128525	KU128529	-	[47]
*G. eickeri*	CMW 49692	MH571690	-	-	[46]
*G. enigmaticum*	Dai 15970	KU572486	-	MG367513	[48]
*G. esculentum*	L4935	MW750242	-	MW839004	[45]
*G. esculentum*	L4946	MW750243	-	-	[45]
*G. gibbosum*	MFLU 19-2176	MN396311	-	MN423118	[43]
*G. hochiminhense*	MFLU 19-2224	MN398324	MN396390	-	[43]
*G. hochiminhense*	MFLU 19-2225	MN396662	MN396391	-	[43]
*G. knysnamense*	CMW 47755	MH571681	-	-	[46]
*G. knysnamense*	CMW 47756	MH571684	-	-	[46]
*G. leucocontextum*	Dai 15601	KU572485	-	MG367516	[49]
*G. leucocontextum*	GDGM 44303	KJ027607	-	-	[14]
*G. lucidum*	Cui 14404	MG279181	-	MG367519	[41]
*G. lucidum*	MFLU 19-2162	MN396341	-	MN423138	[43]
*G. mastoporum*	TNM F0018835	JX840351	-	-	[50]
*G. mastoporum*	TNM-F0018783	JX840352	-	-	[50]
*G. multipileum*	CWN 04670	KJ143913	-	KJ143972	[30]
*G. multipileum*	Dai 9447	KJ143914	-	KJ143973	[30]
*G. myanmarense*	MFLU19-2167	MN396330	MN428672	-	[43]
*G. myanmarense*	MFLU19-2169	-	MN398325	-	[43]
***G. orbiforme***	**MFLUCC 22-0066**	**OP303318**	**OP303348**	**OP407740**	**This study**
*G. orbiforme*	TH15061260	MK345448	-	-	[51]
*G. orbiforme*	GACP14081185	MK313109	-	-	[51]
*G. ryvardenii*	HKAS 58053	HM138671	-	-	[52]
*G. ryvardenii*	HKAS 58054	HM138672	-	-	[52]
*G. shanxiense*	BJTC FM423	MK764268	-	MK783940	[53]
*G. shanxiense*	HSA539	MK764269	-	MK789681	[53]
***G. sichuanense***	**MFLUCC 22-0064**	**MW246109**	**OP303349**	-	**This study**
***G. sichuanense***	**MFLUCC 22-0065**	**MW246111**	**OP303350**	-	**This study**
*G. sichuanense*	B1 1406706	KT693255	-	-	[54]
*G. sichuanense*	Dai3583	JQ781868	-	-	[55]
*G. sichuanense*	Dai12374	JQ781867	-	-	[55]
*G. subflexipes*	Cui 17257	MZ354922	MZ355129	MZ245396	[40]
*G. subflexipes*	Cui 16804	MZ354973	-	MZ345747	[40]
*G. thailandicum*	HKAS104640	MK848681	MK849879	MK875831	[9]
*G. thailandicum*	HKAS104641	MK848682	MK849880	MK875832	[9]
*G. tongshanense*	Cui 17168	MZ354975	MZ355024	-	[40]
*G. tropicum*	Yuan 3490	JQ781880	-	-	[55]
*G. tropicum*	HKAS 97486	MH823539	-	-	[43]
*G. wiiroense*	UMN-20-GHA	KT952361	-	-	[56]
*G. wiiroense*	UMN-21-GHA	KT952363	-	-	[56]
*G. williamsianum*	Dai 16809	MG279183	-	-	[41]
*G. yunlingense*	Cui 16288	MZ354915	MZ355077	-	[40]
*Tomophagus colossus*	TC-02	KJ143923	-	-	[30]

**Table 2:** Effect of media, temperature, pH, and cereal grains media for growing three strains of *Ganoderma* from Thailand.

Parameter		*G. sichuanense*		*G. orbiforme*
		MFLUCC 22-0064	MFLUCC 22-0065	MFLUCC 22-0066
Agar medium	PDA	0.0692 ± 0.0110 ^b^	0.0640 ± 0.0099 ^b^	0.1186 ± 0.0095 ^b^
	PSA	0.1241 ± 0.0029 ^a^	0.1414 ± 0.0217 ^a^	0.0415 ± 0.0124 ^d^
	CMA	0.0313 ± 0.0051 ^c^	0.0567 ± 0.0050 ^bc^	0.0156 ± 0.0079 ^e^
	OMA	0.0363 ± 0.0028 ^c^	0.0392 ± 0.0056 ^c^	0.1496 ± 0.0118 ^a^
	MEA	0.0666 ± 0.0030 ^b^	0.0676 ± 0.0088 ^b^	0.0819 ± 0.0058 ^c^
Temperature (°C)	20	0.0352 ± 0.0033 ^bc^	0.0478 ± 0.0073 ^c^	0.0224 ± 0.0005 ^b^
	25	0.0861 ± 0.0192 ^ab^	0.1282 ± 0.0064 ^b^	0.0707 ± 0.0246 ^a^
	30	0.1295 ± 0.0611 ^a^	0.1721 ± 0.0235 ^a^	0.0512 ± 0.0006 ^a^
	40	0.0028 ± 0.0002 ^c^	0.0021 ± 0.0003 ^d^	0.0005 ± 0.0002 ^b^
pH	2	0.0421 ± 0.0022 ^d^	0.0390 ± 0.0063 ^c^	0.1147 ± 0.0451 ^c^
	4	0.1899 ± 0.0172 ^a^	0.2063 ± 0.0335 ^a^	0.4495 ± 0.0236 ^a^
	6	0.1264 ± 0.0186 ^c^	0.1282 ± 0.0218 ^b^	0.4333 ± 0.0129 ^a^
	7	0.1433 ± 0.0242 ^bc^	0.1807 ± 0.0500 ^a^	0.0575 ± 0.0126 ^c^
	8	0.1570 ± 0.0298 ^b^	0.2221 ± 0.0219 ^a^	0.3185 ± 0.1238 ^b^
Spawn Production	*C. lacryma-jobi*	9.0000 ± 0.5244 ^a^	8.3400 ± 0.5273 ^a^	10.2600 ± 0.5367 ^a^
	*H. vulgare*	7.5600 ± 1.2681 ^a^	6.8000 ± 1.4124 ^a^	10.4000 ± 0.2236 ^a^
	*O. sativa*	10.5000 ± 0.0000 ^a^	8.7200 ± 3.7579 ^a^	8.2200 ± 4.0801 ^a^
	*T. aestivum*	7.8400 ± 2.0852 ^a^	9.2400 ± 1.4690 ^a^	8.3000 ± 0.5431 ^a^
	*Z. mays*	3.7800 ± 4.3672 ^b^	8.2200 ± 3.4745 ^a^	4.2000 ± 1.8574 ^b^

Note: Values are the mean ± SD of the effects of the temperature of the media and the pH were indicated from the growth of dried weight (g) while the effect of the spawn production was performed from the length of the linear mycelium (mm) Number followed by different lowercase letters in column are significantly different according to Duncan’s multiple range test at *p* < 0.05.

**Table 3:** Comparison of the first flush yields of Thai *Ganoderma* species in bag cultivation.

Content	*G. sichuanense*		*G. orbiforme*
	MFLUCC 22-0064	MFLUCC 22-0065	MFLUCC 22-0066
Primordia after inoculation (days)	44–61	35–62	41–58
Average weight (g/bag)	29.43 ± 4.72	33.92 ± 6.98	16.21 ± 3.51
Yield data (g/kg^−1^)	36.79	42.41	20.26
Biological efficiency (B.E.)	132.19 ± 4.72	152.35 ± 6.98	72.79 ± 3.51

**Table 4:** Proximate composition of three wild *Ganoderma* from Thailand (g/100 g of sample).

*Ganoderma* Species	Proximate Analysis (%)			
	Protein	Fat	Fiber	Carbohydrates
MFLUCC 22-0064	12.80 ± 0.15	5.70 ± 0.56	48.61 ± 0.67	4.02 ± 0.11
MFLUCC 22-0065	13.74 ± 0.48	5.11 ± 0.11	47.90 ± 0.39	3.72 ± 0.31
MFLUCC 22-0066	14.67 ± 0.25	4.90 ± 0.56	52.45 ± 0.18	3.16 ± 0.43

**Table 5:** Screening α-glucosidase inhibitory activity of Thai *Ganoderma* extract.

*Ganoderma* Species	Fruiting Bodies		Mycelium	
	EtOH Extract	Water Extract	Acetone Extract	Water Extract
	IC_50_ (µg/mL)		IC_50_ (µg/mL)	
MFLUCC 22-0064	126.94 ± 0.87	inactive	inactive	inactive
MFLUCC 22-0065	171.68 ± 2.78	inactive	inactive	inactive
MFLUCC 22-0066	105.97 ± 1.36	124.40 ± 3.18	inactive	inactive
Acarbose (µg/mL)	168.18 ± 0.89			

## Data Availability

Not applicable.
